# Effects of pioglitazone therapy on blood parameters, weight and BMI: a meta-analysis

**DOI:** 10.1186/s13098-017-0290-5

**Published:** 2017-11-14

**Authors:** Elena Filipova, Katya Uzunova, Krassimir Kalinov, Toni Vekov

**Affiliations:** 1 Science Department, Tchaikapharma High Quality Medicines Inc., 1 G.M. Dimitrov Blvd, 1172 Sofia, Bulgaria; 2 Department of Informatics, New Bulgarian University, 21 Montevideo Str, 1618 Sofia, Bulgaria; 3 Faculty of Pharmacy, Medical University, Pleven, Bulgaria

**Keywords:** Pioglitazone, Glycemic profile, Lipid profile, Weight, BMI

## Abstract

**Background:**

Type 2 diabetes mellitus (T2DM) is one of the most common diseases worldwide and insulin insufficiency and insulin resistance are two main metabolic issues connected with it. The dyslipidemia associated with insulin resistance and T2DM is characterized by higher triglycerides (TGs), higher very-low-density lipoprotein cholesterol and lower apo A1. Pioglitazone, a member of the thiazolidinedione class, with a proven antihyperglycemic effect, is known to positively influence insulin sensitivity and β-cell function and to have the potential to alter the lipid profile.

**Methods:**

The aim of our meta-analysis is to summarize and determine the influence of pioglitazone on the glycemic profile and lipoprotein metabolism as well as on weight and BMI in order to highlight the benefit of pioglitazone therapy in patients with T2DM. A comprehensive literature search was conducted through the electronic databases PubMed, MEDLINE, Scopus, PsyInfo, eLIBRARY.ru (from 2000 until February 2016) to identify studies that investigate the effect of pioglitazone on the glycemic and lipid profile and on the weight and BMI. We chose the random-effects method as the primary analysis. Forest plots depict estimated results from the studies included in the analysis and funnel plots are used to evaluate publication bias. Sensitivity analyses were performed in order to evaluate the degree of influence of the consequent elimination of each individual study on the final result.

**Results:**

Of the 1536 identified sources only 15 randomised trials were included in the meta-analysis. Pioglitazone treatment was associated with improvement in the glycemic profile. It reduced FPG levels by a mean of 1.1–2 mmol/l and HbA1c by a mean of 0.9–1.3%. Our results reaffirmed the hypothesis that pioglitazone has a positive influence on the lipid profile of T2DM patients with increase in TC and HDL, no significant changes in LDL and notable decrease in TGs. Results also showed that pioglitazone therapy led to increase in both weight and BMI (WMD 1.755, 95% CI 0.674 to 2.837 and 1.145, 95% CI 0.389 to 1.901 respectively).

**Conclusion:**

Our results prove that the PPAR γ agonist pioglitazone has the potential to be beneficial to patients with T2DM.

**Electronic supplementary material:**

The online version of this article (10.1186/s13098-017-0290-5) contains supplementary material, which is available to authorized users.

## Background

Type 2 diabetes mellitus (T2DM) is one of the most common diseases worldwide. It is a chronic, metabolic disease characterized by elevated levels of blood glucose, which leads to serious damages to many organs over time. In the past three decades the prevalence of T2DM has risen dramatically in countries of all income levels. World health organization (WHO) statistics showed that there are about 60 million people with diabetes in the European Region, or about 10.3% of men and 9.6% of women aged 25 years and over [1].

Insulin insufficiency and insulin resistance are two main metabolic issues connected with the development of type 2 diabetes. Approximately 92% of patients with type 2 diabetes demonstrate insulin resistance [2, 3]. The dyslipidemia associated with insulin resistance and type 2 diabetes is characterized by higher triglycerides, higher very-low-density lipoprotein (VLDL) cholesterol, lower apo A1, and higher low-density lipoprotein (LDL) particle scores. Diabetes was not associated with elevated LDL cholesterol levels, potentiation of atherogenesis and cardiac dysfunction occurs in the presence of early diabetic symptoms [3–5].

The thiazolidinediones are a class of antidiabetic drugs that exert their action by binding to the peroxisome proliferator-activated receptor gamma (PPAR-γ) [6]. Pioglitazone, a member of this class, with a proven antihyperglycemic effect, is known to positively influence insulin sensitivity and β-cell function and to have the potential to alter the lipid profile [7, 8]. In contrast to the benefits mentioned previously, many authors associate pioglitazone with a significant increase in weight and body mass index (BMI) in patients with T2DM [9–12].

Although the advantages of pioglitazone are well known and outweigh the risks associated with its use many clinicians prefer to prescribe other antihypertensive agents instead. The aim of our meta-analysis is to summarize and determine the influence of pioglitazone on the glycemic profile and lipoprotein metabolism as well as on weight and BMI in order to highlight the benefit of pioglitazone therapy in patients with T2DM.

## Methods

The rationale of this meta-analysis is to determine the effect of pioglitazone therapy on the glycemic and lipid profile in patients with T2DM or impaired glucose tolerance. A comprehensive literature search was conducted through the electronic databases (from 2000 until February 2016) PubMed, MEDLINE, Scopus, PsyInfo, eLIBRARY.ru, as well as registries for data of clinical trials (http://ClinicalTrials.gov and http://www.clinicaltrialsregister.eu) to identify studies that investigate the effect of pioglitazone on the glycemic and lipid profile and on the weight and BMI. The following key words and various combinations were used for the search: pioglitazone; fasting plasma glucose (FBG); glycated hemoglobin (HbA1c); total cholesterol (TC); high-density lipoprotein (HDL); low-density lipoprotein (LDL); triglycerides (TGs); BMI; and weight. Full text articles and abstracts in English and Russian were checked for relevance to the topic and were assessed on the basis of the following inclusion criteria: (1) randomised controlled trials investigating one or more different doses of pioglitazone; (2) determination of changes in the following parameters: FPG, HbA1c, TC, HDL, LDL, TGs, weight and/or BMI throughout treatment with pioglitazone; (3) pioglitazone alone or in combination with any other antidiabetic regimen compared to placebo or active comparators (4) patients with T2DM or impaired glucose tolerance. Any other concomitant conditions related to impaired glucose tolerance or insulin resistance were not considered criteria for exclusion. Inquires were made through contact with authors for any unpublished data, additional unpublished studies or clarifications of methodologies and data included in published articles.

**Fig. 1:**
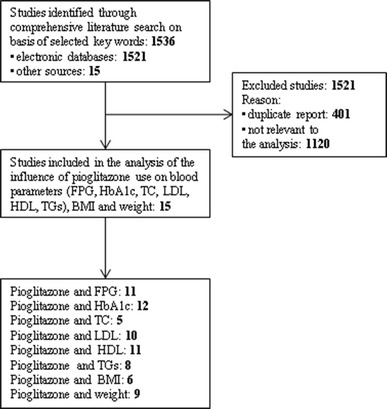
Study selection flow chart

All relevant studies identified were carefully reviewed, sorted, and assessed. Figure 1 depicts the process of selection applied to evaluated studies in order to determine their eligibility for inclusion in the analysis. Extracted data encompassed publication year, duration of treatment, number of patients, FPG (mmol/l), HbA1c (%), TC (mmol/l), LDL (mmol/l), HDL (mmol/l), TGs (mmol/l), BMI (kg/m^2^), weight (kg). Data for all blood parameters as well as BMI and weight was presented as weighed mean difference with a 95% confidence interval (CI).

Due to the significant heterogeneity of the individual studies we chose the random-effects method as the primary analysis. To assess the aforementioned heterogeneity of treatment effect among trials, we used the Cochran Q and the I2 statistics, where p values of less than 0.10 were used as an indication of the presence of heterogeneity and an I2 parameter greater than 50% was considered indicative of substantial heterogeneity. The threshold for statistical significance was set at 0.05. Forest plots depict estimated results from the studies included in the analysis. Funnel plots were used to evaluate publication bias (not shown in the main manuscript, but provided as Additional file 1). Sensitivity analyses were performed in order to evaluate the degree of influence of the consequent elimination of each individual study on the final result. Calculations were made with language for statistical modeling R and MetaXL macro (add-ins of MSExel).

## Results

A large number of studies were identified in the initial search—1536 titles. After evaluation based on the inclusion criteria described in the methods, only 15 studies remained to be included in the analysis of the influence of pioglitazone on blood parameters, BMI and weight (Fig. 1).

Characteristics of the studies included in the meta-analysis are outlined in Table 1. Trial duration lasted from 12 up to 112 weeks. Seven trials were randomised placebo controlled trials [13–18, 24], five trials compared pioglitazone with different antidiabetic treatment [3, 12, 20, 23, 25], and three trials examined the effect of different doses of pioglitazone (from the lowest concentration to the highest) [19, 21, 22].

**Table 1:** Characteristics of studies included in the analysis

Author, year	Study type	Duration of treatment, weeks	Treatment	Number of patients	Characteristics of patients			
					Mean age (years)	Gender (% female)	Mean BMI (kg/m^2^)	Diabetes duration (years)
Berhanu et al. [13]	randomized placebo-controlled	20	PIO + INS	110	52.9	56.4	30.7	7.7
			PLB + INS	112	52.5	58.9	31.8	8.5
Belfort et al. [14]	randomized placebo-controlled	24	PIO 45 mg	26	51	46.1	33.5	NA
			PLB	21	51	70	32.9	NA
Mattoo et al. [15]	randomized, double-blind, prospective, multicenter, placebo-controlled	25	PIO 30 mg + INS	142	58.9	56.7	NA	NA
			PLB + INS	147				
DeFronzo et al. [16]	randomized, double-blind, placebo-controlled	125	PIO initially 30 mg after month 45 mg	303	53	42	33	Patients with IGT and IFGT
			PLB	299	51.5	42	34.5	
DeFronzo et al. [17]	randomized, double-blind, placebo-controlled	125	PIO initially 30 mg after month 45 mg	213	53.9	44	33.4	Patients with IGT and IFGT
			PLB	228	52.5	42	34.4	
Rosenstock et al. [18]	randomised, placebo-controlled study	16	PIO 15 mg + INS	191	56.9	53.9	33.2	NA
			PIO 30 mg + INS	188	57.5	49.5	34.3	NA
			PLB + INS	187	56.7	54.5	33.2	NA
Davidson et al. [19]	randomized, double-blind, multicenter	24	PIO 30 mg + INS	345	NA	NA	33.2	12.9
			PIO 45 mg + INS	345				
Goldberg et al. [3]	randomized, double-blind, prospective, multicenter	24	PIO 30 mg for 12 weeks, then 45 mg for 12 weeks	369	55.9	46.1	33.7	3.9
			ROSI 4 mg for 12 weeks, then 8 mg for 12 weeks	366	56.3	45.1	32.6	4.0
Xu et al. [20]	randomized, multicenter	48	EXE	149	NA	NA	NA	NA
			INS	149				
			PIO	118				
Bolli et al. [12]	multicentre, randomized, active-controlled	52	PIO 30 mg + MET 1500 mg	281	57	35.9	32.1	6.4
			VIL 100 mg + MET 1500 mg	295	56.3	38.3	32.2	6.4
Gerber et al. [21]	randomized, multicenter	20	PIO 30 mg	76	NA	NA	NA	NA
			PIO 30 mg for 12 weeks then 45 mg for 8 weeks	74				
			PIO 45 mg	84				
Panikar et al. [22]	randomised	24	PIO 7.5 mg	77	58.2	47.4	27.6	< 2
			PIO 15 mg	80	56.6	43.7	26.6	
			PIO 45 mg	80	53.7	40	25.8	
Kodama et al. [23]	prospective, randomized, open-label, comparator-controlled	16	PIO	32	68.4	28.1	25.2	NA
			GLI	21	66.7	19	24.7	
Shah et al. [24]	randomised	12–16	INS + PIO 45 mg	12	58	16	36.7	NA
			INS + PLB	13				
Yoshii et al. [25]	prospective, randomized, open-label, multicenter	96	with PIO	234	69.0	36.8	24.2	11.1
			without PIO	247	68.9	34.0	24.3	11.5

Mattoo et al. [15], Berhanu et al. [13], Shah et al. [24] and Rosenstock et al. [18] investigated the metabolic effects of pioglitazone as an add-on treatment to insulin by determining HbA1c, FPG, HDL, LDL and TGs. Belfort et al. [14] examined pioglitazone’s effect over nonalcoholic steatohepatitis. DeFronzo et al. [16, 17] contemplated the use of pioglitazone for T2DM prevention in IGT and its influence on β-cell function. Davidson et al. [19], Gerber et al. [21] and Panikar et al. [22] discussed the dose related response to pioglitazone. Active comparator studies by Goldberg et al. [3], Bolli et al. [12], Xu et al. [20] and Yoshii et al. [25] compared the effect of pioglitazone to that of rosiglitazone, vildagliptin, exenatide and insulin, and other antihyperglycemic drugs, respectively. Kodama et al. [23] evaluated visceral fat metabolism in patients with impaired glucose tolerance or T2DM treated with pioglitazone.

The 15 studies that satisfied the inclusion criteria were included in the analysis of the influence of pioglitazone on the glycemic and lipid profile of patients with T2DM as well as on changes in weight and BMI. Pooled results showed that pioglitazone use was associated with an increased likelihood for improvement in the following blood parameters: FPG, HbA1c, HDL, LDL. Changes in TC and TGs were not statistically significant while weight and BMI increase significantly (Table 2).

**Table 2:** Influence of pioglitazone on glycaemic and lipid profile, weight and BMI in patients with T2DM

Author, year	FPG* (mmol/l)	HbA1c* (%)	Total cholesterol* (mmol/l)	LDL* (mmol/l)	HDL* (mmol/l)	Triglycerides* (mmol/l)	BMI* (kg/m^2^)	Weight* (kg)
Berhanu et al. [13]	0.47 (− 0.14; 1.09)	− 1.60 (− 1.82; − 1.38)	0.15 (0.01; 0.27)	0.10 (− 0.02; 0.22)	0.11 (0.07; 0.15)	− 0.002 (− 0.22; 0.22)		
Belfort et al. [14]	− 1.11 (− 1.93; − 0.29)	− 0.70 (− 1.35; − 0.05)	0.13 (− 0.36; 0.61)	0.05 (− 0.41; 0.52)	0.08 (− 0.05; 0.20)	− 0.27 (− 0.80; 0.25)	1.1 (− 1.79; 3.99)	2.5 (− 7.97; 12.97)
DeFronzo et al. [16]	− 0.59 (− 0.67; − 0.50)				0.50 (0.49; 0.51)	− 1.1 (− 1.17; − 1.03)	1.4 (1.32; 1.48)	3.8 (3.60; 4.00)
DeFronzo et al. [17]	− 0.22 (− 0.23; − 0.21)						1.6 (1.21; 1.99)	
Mattoo et al. [15]	− 1.22 (− 2.24; − 0.20)	− 0.74 (− 1.01; − 0.47)		− 0.2 (− 0.24; 0.20)	0.12 (0.04; 0.19)			
Rosenstock et al. (15 mg) [18]	− 1.91 (− 2.46; − 1.37)	− 0.99 (− 1.15; − 0.83)		0.13 (0.04; 0.22)	0.18 (0.10; 0.26)	0.06 (− 0.09; 0.21)		
Rosenstock et al. (30 mg) [18]	− 2.67 (− 3.22; − 2.12)	− 1.26 (− 1.42; − 1.10)		0.07 (− 0.02; 0.02)	0.24 (0.16; 0.32)	− 0.12 (− 0.26; 0.03)		
Davidson et al. (30 mg) [19]	− 1.77 (− 2.21; − 1.33)	− 1.20 (− 1.36; − 1.04)		0.17 (0.08; 0.25)	0.25 (0.19; 0.31)	− 0.05 (− 0.12; 0.01)		
Davidson et al. (45 mg) [19]	− 2.54 (− 2.98; − 2.11)	− 1.50 (− 1.66; − 1.34)		0.17 (0.08; 0.26)	0.34 (0.27; 0.40)	− 0.07 (− 0.13; − 0.002)		
Goldberg et al. [3]	− 1.84 (− 2.08; − 1.60)	− 0.70 (− 0.90; − 0.50)	0.23 (0.13; 0.32)	0.32 (0.24; 0.40)	0.13 (0.11; 0.16)	− 0.59 (− 0.76; − 0.41)		2.0 (1.64; 2.39)
Panikar et al. (7.5 mg) [22]		− 1.24 (− 2.51; 0.03)					0.88 (− 4.55; 6.31)	0.33 (− 1.65; 2.31)
Panikar et al. (15 mg) [22]		− 1.18 (− 2.34; − 0.02)					1.62 (− 4.08; 7.32)	0.62 (− 1.54; 2.78)
Panikar et al. (30 mg) [22]		− 1.25 (− 2.90; 0.40)					2.72 (− 3.10; 8.54)	1.03 (− 1.20; 3.26)
Xu et al. [20]	− 2.00 (− 2.39; − 1.61)	− 1.50 (− 1.70; − 1.30)	− 0.10 (− 0.30; 0.10)	− 0.10 (− 0.30; 0.10)	0.16 (0.12; 0.20)	− 0.20 (− 0.40; − 0.004)	0.00 (− 0.20; 0.20)	0.00 (− 0.78; 0.78)
Yoshii et al. [25]				− 0.22 (− 0.33; − 0.08)	0.12 (0.05; 0.19)			
Bolli et al. [12]	− 1.60 (− 1.95; − 1.33)	− 0.60 (− 0.71; − 0.45)						2.60 (2.01; 3.19)
Kodama et al. [23]		− 0.36 (− 0.73; 0.01)		− 0.01 (− 0.34; 0.32)	0.14 (− 0.03; 0.31)			0.60 (− 5.45; 6.65)
Shah et al. [24]		− 0.5 (− 0.77; − 0.23)					1.5 (1.11; 1.89)	4.9 (− 3.92; 13.72)
Gerber et al. (30 mg) [21]	− 2.6 (− 1.39; − 0.90)	− 1.1 (− 3.26; 1.06)	− 0.13 (− 1.87; 1.61)	− 0.01 (− 1.56; 1.54)	0.11 (− 0.68; 0.68)	− 0.87 (− 3.03; 4.77)		2.6 (− 2.89; 8.09)
Gerber et al. (30/45 mg) [21]	− 1.9 (− 1.38; − 0.73)	− 1.1 (− 3.84; 1.64)	− 0.03 (− 2.62; 2.56)	− 0.12 (− 1.69; 1.45)	0.12 (− 0.55; 0.79)	− 0.60 (− 4.56; 5.16)		2.7 (− 3.18; 8.58)
Gerber et al. (45 mg) [21]	− 1.5 (− 1.23; − 0.52)	− 0.9 (− 4.04; 2.24)		− 0.12 (− 1.61; 1.37)	0.08 (− 0.37; 0.53)	− 0.28 (− 3.28; 2.72)		2.8 (− 3.67; 9.27)

* Weighted mean difference (95% CI)

## Pioglitazone and glycemic profile

**Fig. 2:**
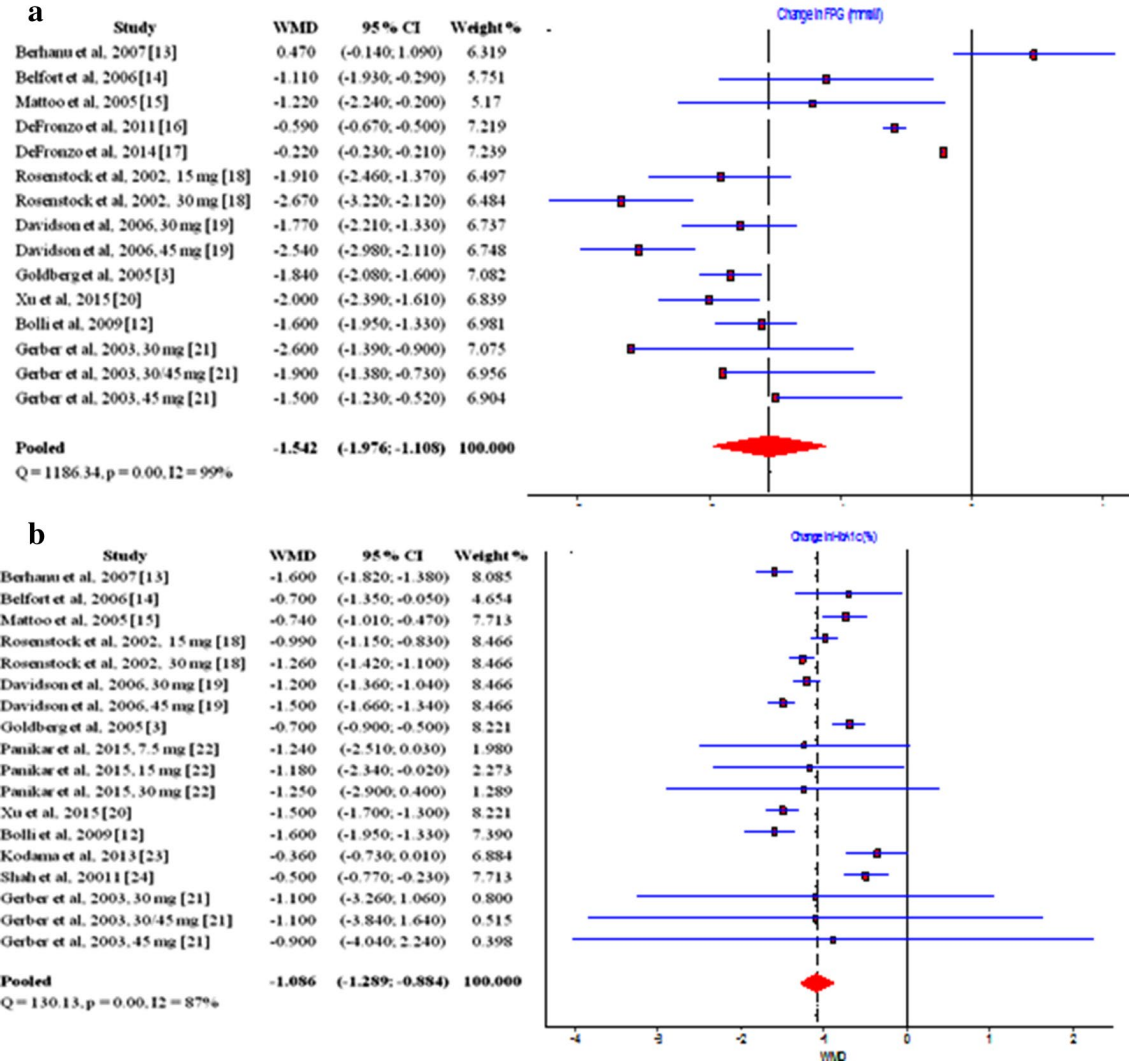
Influence of pioglitazone on the glycemic profile in T2DM patients, **a** influence of pioglitazone on the FPG; **b** influence of pioglitazone on the HbA1c

Figure 2 depicts the estimated effect of pioglitazone on the glycemic profile in T2DM patients. A total of eleven studies [3, 12–21] reporting data for 4812 patients of whom 2859 were treated with pioglitazone were used to estimate the effects of pioglitazone therapy on FPG levels. The pooled results provided a WMD of—1.542 with a 95% CI (− 1.976; − 1.108) suggesting a significant decrease in FPG values (Fig. 2a). Results from twelve studies [3, 12–15, 18–24] with 2630 pioglitazone treated patients provided evidence for the effects of pioglitazone therapy on HbA1c levels. The summary WMD was − 1.086 with 95% CI (− 1.289; − 0.884) (Fig. 2b). These results speak in favor of the positive impact of pioglitazone therapy with a significant reduction in HbA1c. All in all, pioglitazone treatment was associated with improvement in the glycemic profile. It reduced FPG levels by a mean of 1.1–2 mmol/l and HbA1c by a mean of 0.9–1.3%.

## Pioglitazone and lipid profile

**Fig. 3:**
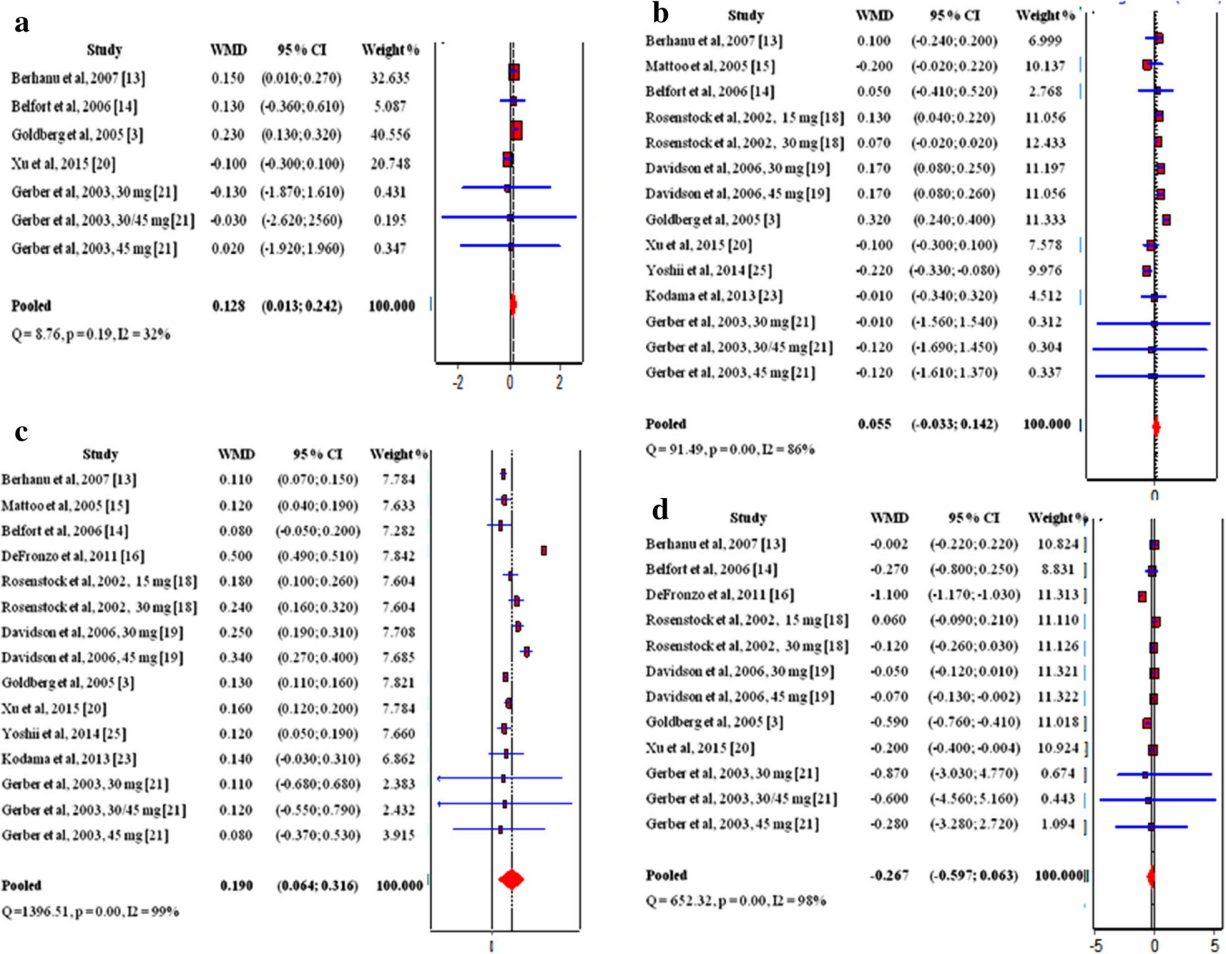
Influence of pioglitazone on the lipid profile in T2DM patients, **a** influence of pioglitazone on the TC; **b** influence of pioglitazone on the LDL; **c** influence of pioglitazone on the HDL; **d** influence of pioglitazone on theTGs

Data from five studies [3, 13, 14, 20, 21] with 884 patients treated with pioglitazone and 797 controls were used in the estimation of the effect of pioglitazone on TC levels. Pooled results showed a slight increase in TC levels WMD 0.128 (0.013; 0.24) (Fig. 3a). A total of 10 studies [3, 13–15, 18–21, 23, 25] presenting data for 2334 pioglitazone users produced a WMD 0.055 (− 0.033; 0.142) in LDL levels designating a non significant change compared to baseline (Fig. 3b). Results from 11 studies [3, 13–16, 18–21, 23, 25] providing data for 2637 pioglitazone treated patients were pooled to estimate the effect of pioglitazone on HDL levels. WMD 0.190 (0.064; 0.316) showed that HDL levels increased in pioglitazone treated patients (Fig. 3c). Pooled WMD from 8 studies [3, 13, 14, 16, 18–21] investigating the effect of pioglitazone on TGs demonstrated its tendency to decrease TGs levels compared to baseline (Fig. 3d). Our results reaffirm the hypothesis that pioglitazone has a positive influence on the lipid profile of T2DM patients with increase in TC and HDL, no significant changes in LDL and notable decrease in TGs.

## Pioglitazone, weight and BMI

**Fig. 4:**
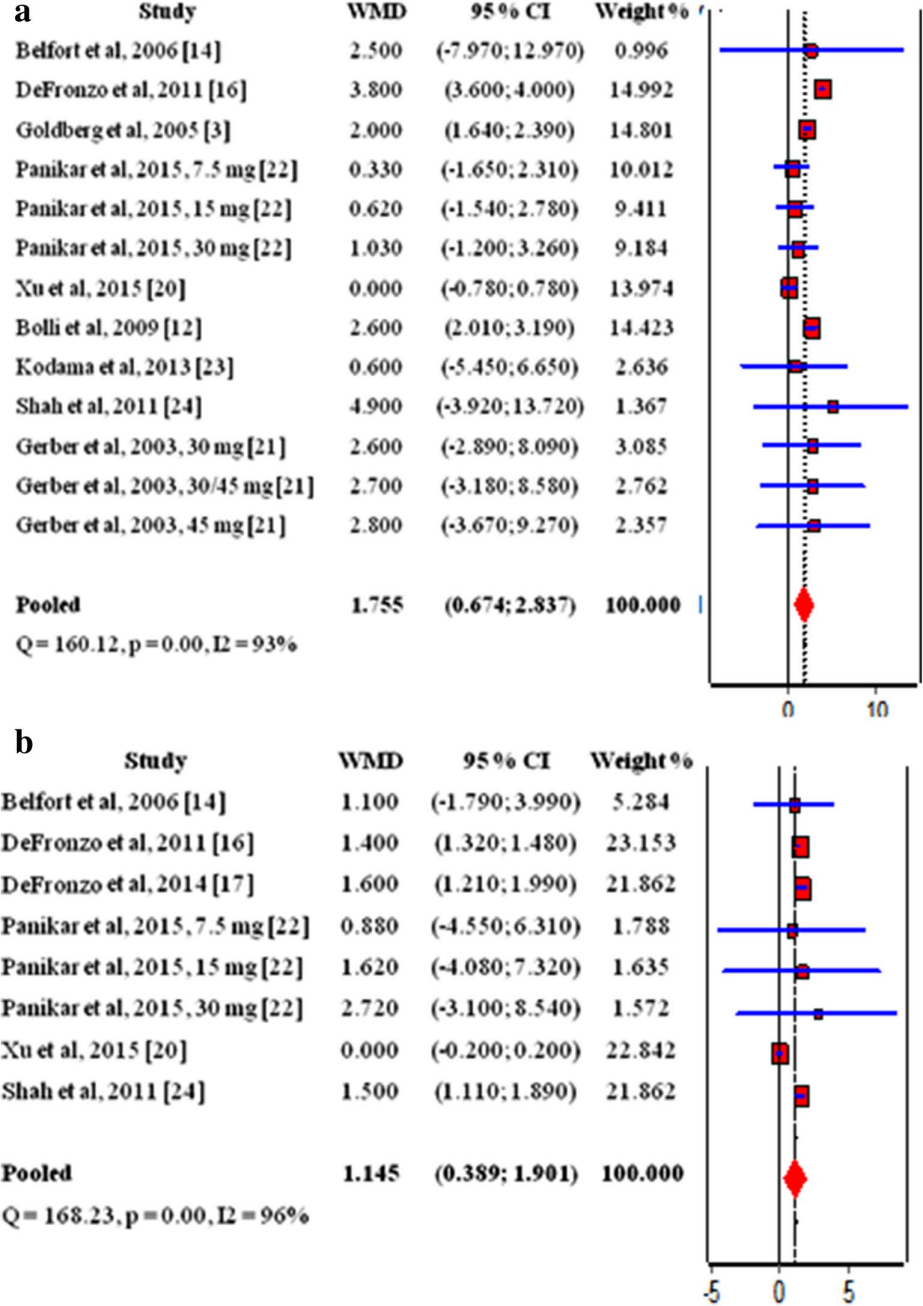
Influence of pioglitazone on the weight and BMI in T2DM patients, **a** influence of pioglitazone on the weight; **b** influence of pioglitazone on the BMI

Nine studies [3, 12, 14, 16, 20–24] and 6 studies [14, 16, 17, 20, 22, 24] were evaluated in the determination of the effect of pioglitazone on the weight and BMI in T2DM patients, respectively. Results showed that pioglitazone therapy led to increase in both weight and BMI (WMD 1.755, 95% CI 0.674 to 2.837 and 1.145, 95% CI 0.389 to 1.901 respectively) (Fig. 4a, b).

## Sensitivity analysis

The results from the statistical analyses for all studies in relation to the investigated blood parameters, weight and BMI are summarized and presented in Table 3. When each study was subsequently excluded from the analysis, pooled WMD for FPG are in the range − 1.678 to − 1.462 and for HbA1c − 1.138 to − 1.041. The lack of substantial changes in WMD suggests consistency of our findings and confirms the positive effect of pioglitazone on the glycemic profile. When parameters measured in order to estimate the influence of pioglitazone on the lipid profile are subjected to sensitivity analysis pooled WMD for TC is in the range 0.077 to 0.199, for LDL—0.024 to 0.088, for HDL—0.172 to 0.199 and for TGs: − 0.300 to − 0.136. They confirm our findings and signify of positive effect of pioglitazone on the lipid profile. Pooled results for BMI and weight also suggest consistency of our findings.

**Table 3:** Sensitivity analysis

Excluded study	Pooled WMD for FPG (mmol/l)	Pooled WMD for HbA1c (%)	Pooled WMD for total cholesterol (mmol/l)	Pooled WMD for LDL (mmol/l)	Pooled WMD for HDL (mmol/l)	Pooled WMD for triglycerides (mmol/l)	Pooled WMD for BMI (kg/m^2^)	Pooled WMD for weight (kg)
Berhanu et al. [13]	− 1.678 (− 2.128; − 1.228)	− 1.041 (− 1.248; − 0.835)	0.091 (− 0.111; 0.294)	0.051 (− 0.042; 0.143)	0.197 (0.067; 0.327)	− 0.300 (− 0.653; 0.054)	1.148 (0.368; 1.928)	1.747 (0.657; 2.837)
Belfort et al. [14]	− 1.568 (− 2.016; − 1.121)	− 1.105 (− 1.312; − 0.897)	0.121 (− 0.011; 0.253)	0.054 (− 0.035; 0.144)	0.199 (0.068; 0.329)	− 0.267 (− 0.614; 0.080)		
DeFronzo et al. [16]	− 1.608 (− 2.284; − 0.933)				0.172 (0.131; 0.213)	− 0.136 (− 0.244; − 0.028)	1.091 (0.060; 2.121)	1.396 (0.559; 2.233)
DeFronzo et al. [17]	− 1.642 (− 2.152; − 1.131)						1.026 (0.108; 1.944)	
Mattoo et al. [15]	− 1.560 (− 2.005; − 1.114)	− 1.115 (− 1.324; − 0.906)		0.086 (0.001; 0.171)	0.196 (0.065; 0.327)			
Rosenstock et al. (15 mg) [18]	− 1.517 (− 1.962; − 1.071)	− 1.093 (− 1.316; − 0.870)		0.043 (− 0.056; 0.143)	0.191 (0.059; 0.323)	− 0.308 (− 0.665; 0.049)		
Rosenstock et al. (30 mg) [18]	− 1.464 (− 1.902; − 1.026)	− 1.068 (− 1.295; − 0.842)		0.046 (− 0.077; 0.169)	0.186 (0.054; 0.318)	− 0.286 (− 0.649; 0.077)		
Davidson et al. (30 mg) [19]	− 1.526 (− 1.971; − 1.080)	− 1.074 (− 1.301; − 0.847)		0.039 (− 0.060; 0.137)	0.185 (0.051; 0.319)	− 0.296 (− 0.687; 0.095)		
Davidson et al. (45 mg) [19]	− 1.470 (− 1.905; − 1.036)	− 1.048 (− 1.258; − 0.838)		0.039 (− 0.059; 0.137)	0.177 (0.043; 0.312)	− 0.293 (− 0.689; 0.103)		
Goldberg et al. [3]	− 1.520 (− 1.954; − 1.086)	− 1.122 (− 1.325; − 0.920)	0.077 (− 0.029; 0.183)	0.024 (− 0.057; 0.104)	0.196 (0.066; 0.325)	− 0.228 (− 0.585; 0.130)		1.708 (0.364; 3.053)
Panikar et al. (7.5 mg) [22]		− 1.083 (− 1.288; − 0.877)					1.150 (0.385; 1.915)	1.915 (0.786; 3.043)
Panikar et al. (15 mg) [22]		− 1.084 (− 1.290; − 0.878)					1.137 (0.373; 1.902)	1.873 (0.743; 3.004)
Panikar et al. (30 mg) [22]		− 1.084 (− 1.289; − 0879)					1.120 (0.356; 1.884)	1.829 (0.649; 2.963)
Xu et al. [20]	− 1.509 (− 1.950; − 1.067)	− 1.049 (− 1.261; − 0.837)	0.199 (0.124; 0.275)	0.067 (− 0.024; 0.158)	0.192 (0.059; 0.326)	− 0.276 (− 0.633; 0.082)	1.412 (1.335; 1.488)	2.097 (1.101; 3.092)
Yoshii et al. [25]				0.088 (0.003; 0.172)	0.196 (0.065; 0.327)			
Bolli et al. [12]	− 1.538 (− 1.984; − 1.092)	− 1.045 (− 1.254; − 0.836)						1.613 (0.341; 2.885)
Kodama et al. [23]		− 1.141 (− 1.339; − 0.943)		0.057 (− 0.033; 0.148)	0.194 (0.063; 0.324)			1.786 (0.689; 2.884)
Shah et al. [24]		− 1.138 (− 1.334; − 0.941)					1.054 (0.132; 1.977)	1.711 (0.619; 2.803)
Gerber et al. (30 mg) [21]	− 1.462 (1.857; − 1.067)	− 1.086 (− 1.290; − 0.882)	0.124 (− 0.001; 0.249)	0.055 (− 0.034; 0.143)	0.192 (0.065; 0.320)	− 0.263 (− 0.595; 0.068)		1.728 (0.625; 2.830)
Gerber et al. (30/45 mg) [21]	− 1.516 (− 1.956; − 1.075)	− 1.086 (− 1.290; − 0.882)	0.123 (− 0.003; 0.248)	0.055 (− 0.033; 0.143)	0.192 (0.065; 0.319)	− 0.266 (− 0.597; 0.065)		1.728 (0.627; 2.828)
Gerber et al. (45 mg) [21]	− 1.545 (− 1.993; − 1.097)	− 1.087 (− 1.290; − 0.883)	0.123 (− 0.003; 0.249)	0.055 (− 0.033; 0.143)	0.195 (0.066; 0.323)	− 0.267 (− 0.599; 0.065)		1.729 (0.632; 2.827)

## Discussion

TZDs as a whole and pioglitazone in particular are known to favorably influence the majority of the components of insulin resistance characteristic of T2DM, like adiposity, dyslipidaemia, hyperglycaemia, hypertension, cardiovascular abnormalities, hyper coagulation, vasculopathy, accelerated atherosclerosis, and changes in liver and ovaries [26]. A lot of authors conclude that pioglitazone successfully reduces HbA1c as monotherapy and in combination compared to placebo and other antihyperglycemic agents [10, 11, 27–33]. Reported data has indicated that the probability of reaching target HbA1c < 7% is higher in the case of therapy with pioglitazone [34]. Pioglitazone has a manageable safety profile but remains associated with weight gain and edema [35]. Nevertheless, pioglitazone is not the drug of choice for many clinicians.

The main aim of our meta-analysis was to follow up changes in metabolic parameters and to evaluate the influence of pioglitazone on the glycemic and lipid profile of patients with T2DM. We reviewed a large number of sources and based our conclusions on articles we deemed to be of satisfactory quality.

Our results indicated that pioglitazone use was associated with positive effect on the glycemic profile with significant reductions in FPG and HbA1c (see Fig. 1a, b). Comparison with other sources showed consistency of our findings with published results. For example Lu et al. [27] and Scherbaum et al. [28] reported statistically significant reductions of FPG and HbA1c of around − 1.48 to − 2 mmol/l and − 0.92 to − 1.05% respectively when pioglitazone therapy was compared to placebo. Other authors have reported similar or even larger reductions in FPG and HbA1c registered when pioglitazone treatment was compared to other antidiabetic drugs as monotherapy or in combination. Russel-Jones et al. [10] reported reductions of − 2.6 mmol/l and − 1.63% for FPG and HbA1c respectively with pioglitazone against treatment with sitagliptin, metformin or exenatide. When pioglitazone treatment was combined with the use of metformin, reductions of FPG and HbA1c were − 1.7 mmol/l and − 0.74% respectively, according to Chawla et al. [30]. Same authors reported decrease of FPG with sitagliptin/metformin combination of − 1.1 mmol/l suggesting more pronounced effect of pioglitazone despite the lack of statistical significance of the between group difference. Kaur et al. [36] concluded that pioglitazone combined with metformin and sulphonylurea produced a greater reduction in HbA1c and the results were statistically significant.

Apart from the achievement of a satisfactory glycemic profile and improvement of the insulin sensitivity pioglitazone use is known to be associated with the amelioration of dyslipidemia in patients with T2DM [3, 37]. Khan et al. [38] performed an open-label, randomized comparison of rosiglitazone and pioglitazone in patients previously treated with troglitazone. In that study, conversion to pioglitazone was associated with significant improvements in all lipid parameters. In a retrospective review of randomly selected medical records, it was shown that treatment with PIO was associated with greater beneficial effects on blood lipid profile with a reduction in mean TGs of 0.62 mmol/l, a reduction in TC of 0.22 mmol/l, an increase in HDL of 0.068 mmol/l, and a reduction in LDL-C of 0.13 mmol/l [39].

In contrast to the aforementioned benefits, the TZDs, in particular pioglitazone increased body weight [9–12, 31] in part because of differentiation of adipocytes and expansion of adipocyte mass. Activation of PPAR-γ stimulates differentiation to insulin-sensitive smaller adipocytes and redistributes fat from visceral to subcutaneous depots, a pattern that has been associated with lower cardiovascular disease (CVD) risk. A recent meta-analysis incorporating results from 10 randomised trials concludes that pioglitazone lowers the risk of recurrent major adverse cardiovascular events, stroke, or myocardial infarction in patients with clinical manifest vascular disease [40]. A critical and updated overview of the main glucose-lowering agents and their risk/benefit ratio for the prevention of CVD in patients with T2DM speaks in favour of the positive effect of pioglitazone on the lipid profile and its ability to reduce the CVD risk [41]. Pioglitazone treatment also reduces visceral fat and decrease liver-fat resulting in increased insulin sensitivity in these tissues [23, 24].

Our study has limitations which are mainly connected with the degree of differentiation of parameters estimated in the studies included in the analysis. This is supposedly one of the reasons for the heterogeneity that we noted. Other factors it could be attributed to are the variation in the duration of therapies, different aspects of studies, difference in the antidiabetic drug experience of patients and the type of anidiabetic drugs used concomitantly with pioglitazone. We did not have individual patient data and therefore could not evaluate other factors that could potentially influence the glycemic or lipid profile.

## Conclusion

Many clinicians worldwide are inclined to avoid pioglitazone use for the treatment of patients with T2DM. By summarizing and analyzing data from numerous trials we have been able to highlight the beneficial and unambiguous effect of pioglitazone on the glycemic profile, characterized by considerable reductions in the FPG and HbA1c. Another positive trend that we have determined is the improvement of the lipid balance. These conclusions are not contradictory to what has already been published by other authors and are a prerequisite for wider application of pioglitazone in the clinical practice. The only drawback we estimated was the increase of BMI and weight in patients with T2DM although we believe it should not discourage clinicians to provide diabetes patients the opportunity to exploit the benefits of the antihyperglycemic medication pioglitazone.

## Additional file

**Additional file 1: Figure S1.** Funnel plot for FPG. **Figure S2.** Funnel lot for HbA1c. **Figure S3.** Funnel plot for total cholesterol. **Figure S4.** Funnel plot for LDL. **Figure S5.** Funnel plot for HDL. **Figure S6.** Funnel plot for TGs. **Figure S7.** Funnel plot for weight. **Figure S8.** Funnel plot for BMI.

## Abbreviations

T2DM: type 2 diabetes mellitus
TZDs: thiazolidinediones
PPARγ: peroxisome proliferator-activated receptors gamma
HbA_1c_: glycated hemoglobin
FPG: fasting plasma glucose
HDL: high-density lipoprotein
LDL: low-density lipoprotein
TGs: triglycerides
TC: total cholesterol
BMI: body mass index
WMD: weighted mean difference
CVD: cardiovascular disease
CI: confidence interval
