# Supplementary material for: Effects of pioglitazone therapy on blood parameters, weight and BMI: a meta-analysis
Source: Diabetol Metab Syndr. 2017 Nov 14;9:90. doi: 10.1186/s13098-017-0290-5 (PMC5686837; doi:10.1186/s13098-017-0290-5)
Supplement: Supplementary file 1 — Additional file 1: Figure S1. Funnel plot for FPG. Figure S2. Funnel lot for HbA1c. Figure S3. Funnel plot for total cholesterol. Figure S4. Funnel plot for LDL. Figure S5. Funnel plot for HDL. Figure S6. Funnel plot for TGs. Figure S7. Funnel plot for weight. Figure S8. Funnel plot for BMI. [file 13098_2017_290_MOESM1_ESM.pdf]

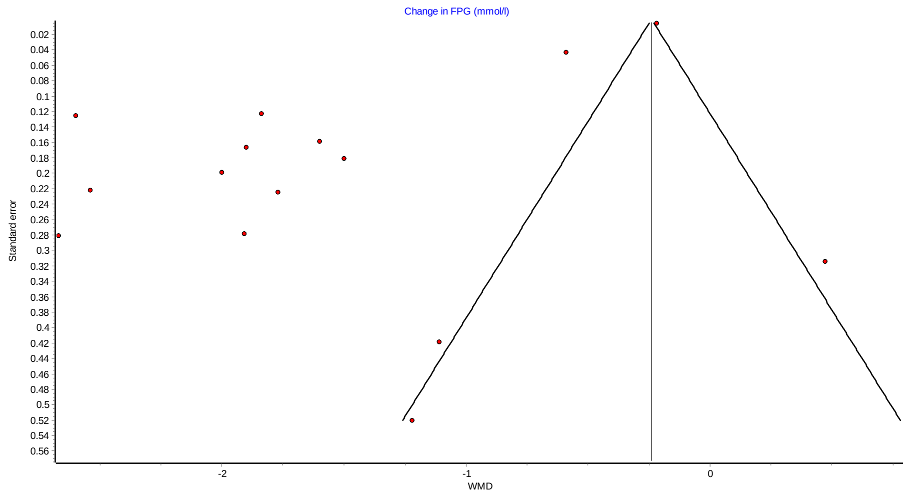

Figure S1 Funnel plot for FPG

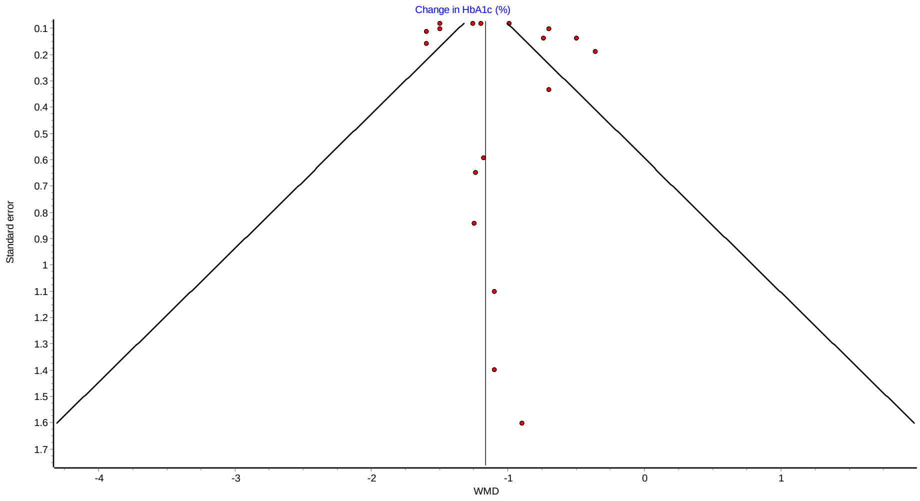

Figure S2 Funnel lot for HbA1c

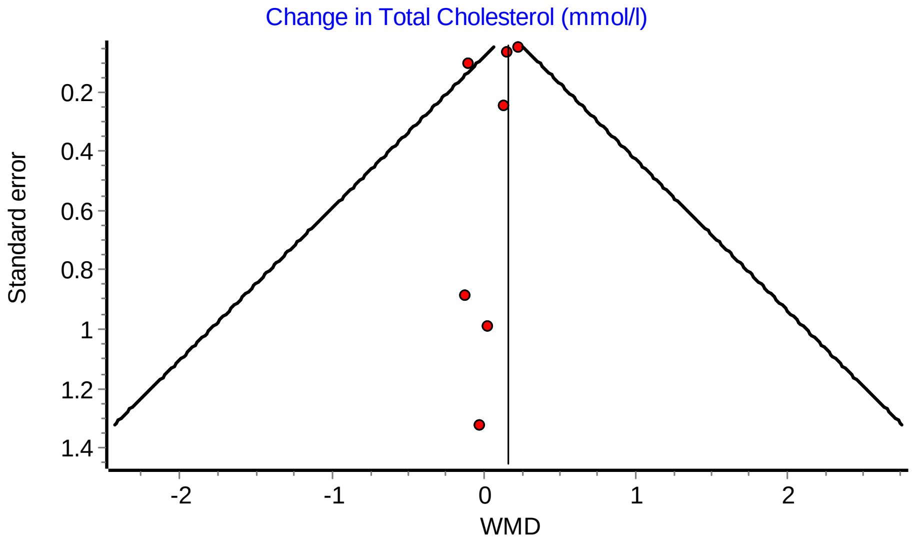

Figure S3 Funnel plot for total cholesterol

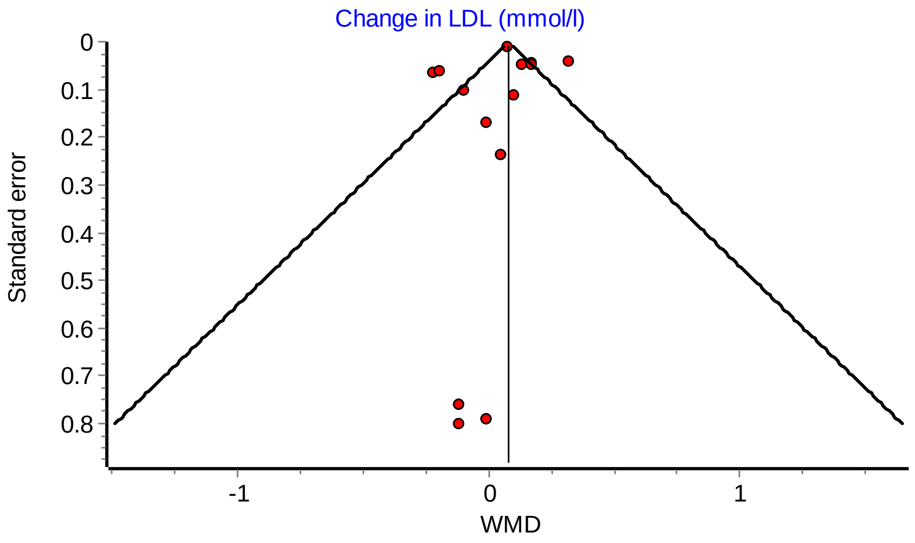

Figure S4 Funnel plot for LDL

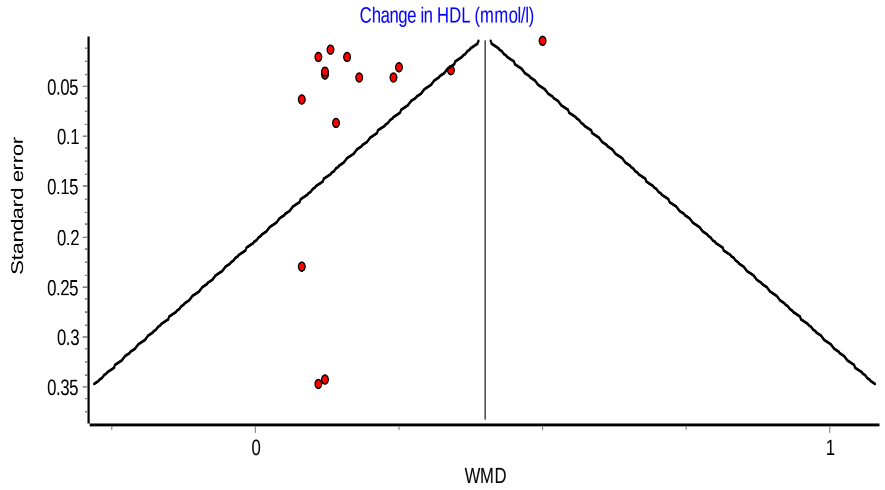

Figure S5 Funnel plot for HDL

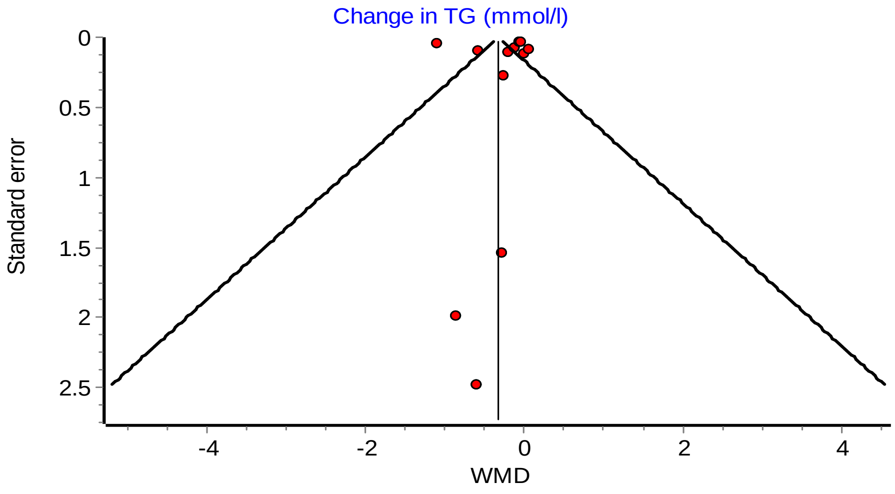

Figure S6 Funnel plot for TGs

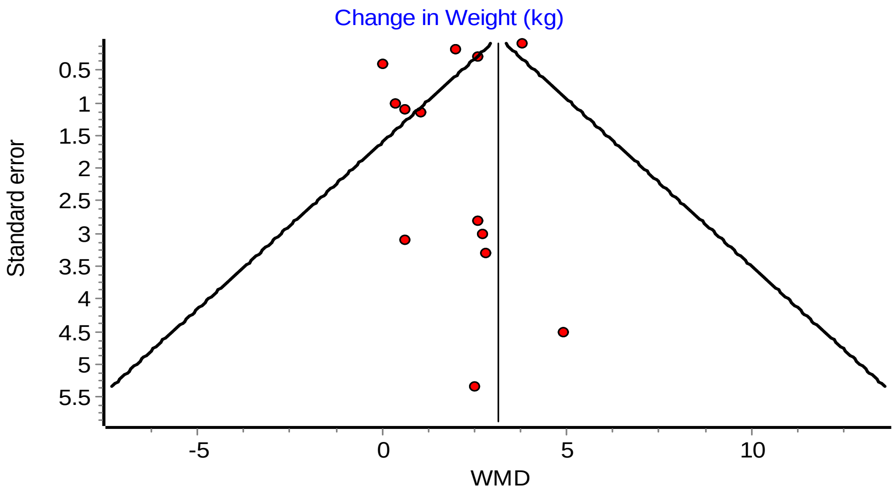

Figure S7 Funnel plot for weight

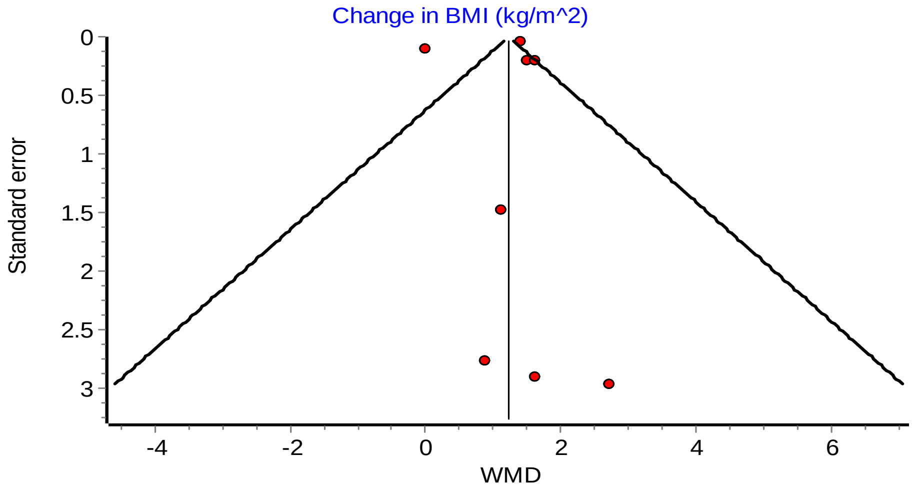

Figure S8 Funnel plot for BMI
